# The Combined Effects of Obesity, Abdominal Obesity and Major Depression/Anxiety on Health-Related Quality of Life: the LifeLines Cohort Study

**DOI:** 10.1371/journal.pone.0148871

**Published:** 2016-02-11

**Authors:** Yeshambel T. Nigatu, Sijmen A. Reijneveld, Peter de Jonge, Elisabeth van Rossum, Ute Bültmann

**Affiliations:** 1 Department of Health Sciences, University Medical Center Groningen, University of Groningen, Groningen, The Netherlands; 2 Interdisciplinary Center Psychopathology and Emotion Regulation, Department of Psychiatry, University Medical Center Groningen, University of Groningen, Groningen, the Netherlands; 3 Department of Internal Medicine, Erasmus Medical Center, Rotterdam, the Netherlands; Charité-Universitätsmedizin Berlin, Campus Benjamin Franklin, GERMANY

## Abstract

**Background:**

Obesity and major depressive disorder (MDD)/anxiety disorders often co-occur and aggravate each other resulting in adverse health-related outcomes. As little is known about the potential effects of interaction between obesity and MDD and/or anxiety disorders on health-related quality of life (HR-QoL), this study was aimed at examining these combined effects.

**Methods:**

We collected data among N = 89,332 participants from the LifeLines cohort study. We categorized body weight using body mass index (kg/m^2^) as normal weight (18.5–24.99), overweight (25–29.9), mild obesity (30–34.9) and moderate/severe obesity (≥ 35); we measured abdominal obesity using a waist circumference of ≥102 and ≥ 88 cm for males and females, respectively. MDD and anxiety disorders were diagnosed with the Mini-International Neuropsychiatric Interview. HR-QoL was assessed using the RAND-36 questionnaire to compute physical and mental quality of life scores. We used binary logistic and linear regression analyses.

**Results:**

The combined effect of obesity and MDD and/or anxiety disorders on physical QoL was larger than the sum of their separate effects; regression coefficients, B (95%-confidence interval, 95%-CI) were: - 1.32 (-1.75; -0.90). However, the combined effect of obesity and major depression alone on mental QoL was less than the additive effect. With increasing body weight participants report poorer physical QoL; when they also have MDD and/or anxiety disorders participants report even poorer physical QoL. In persons without MDD and/or anxiety disorders, obesity was associated with a better mental QoL.

**Conclusions:**

Obesity and MDD and/or anxiety disorders act synergistically on physical and mental QoL. The management of MDD and/or anxiety disorders and weight loss may be important routes to improve HR-QoL.

## Introduction

Obesity, major depressive disorder (MDD) and anxiety disorders are major public health problems, posing enormous challenges for the decades to come [1,2]. Obesity and MDD and/or anxiety disorders are associated with long-term disability, morbidity and mortality, and enormous economic costs [3–5]. Since the 1980s the prevalence of obesity has tripled in many countries of the World Health Organization (WHO) European Region, and continues to rise at an alarming rate. The rate of MDD and/or anxiety disorders has also increased in the past decade. For instance, in the UK, the incidence of depressive symptoms rose threefold from the baseline of 5.11/1000 person years in 1996 to 15.5/1000 person years in 2006 [4,6]. MDD is even expected to be one of the top leading causes of disability-adjusted life years in 2030 [7]. Obesity and MDD and/or anxiety have become the most serious health risks today, and are associated with major chronic diseases such as cardiovascular diseases, type 2 diabetes, orthopedic problems and certain kinds of cancer [8,9].

Co-occurrence of chronic physical conditions with MDD and/or anxiety disorders may have even worse consequences, including a poorer health-related quality of life (HR-QoL) [10]. HR-QoL has gained increasing interest as an outcome measure in clinical practice and public health settings [11]. Alley et al showed that obesity is associated with poor QoL, especially due to its earlier age of onset and long-term exposure [12]. MDD and/or anxiety disorders are also associated with significant reductions in HR-QoL [4]. During a depressive episode, a patient’s level of HR-QoL is the same as that of as a patient with a severe stroke [13].

Several studies have reported that obesity and MDD and/or anxiety disorders often co-occur and are bidirectionally inter-related [14–16]. However, obesity and MDD and/or anxiety disorders have more often been considered separate conditions without taking into account their potential interaction on HR-QoL. For example, the effect of general and abdominal obesity on HR-QoL may further increase in persons with MDD and/or anxiety disorders compared to those without such disorders. Regarding this, the general obesity reflects excess total body fat whereas abdominal obesity in particular reflects excess visceral fat which has been suggested to be in particular deleterious [17]. However, for both obesity and abdominal obesity little is known about the potential effects of their interaction with MDD and/or anxiety disorders on HR-QoL. The main reasons to examine their interaction or combined effect on HR-QoL are: 1) obesity and MDD are bidirectionally related and overlapping risk factors for major chronic diseases [14–16,18]; 2) patients with obesity and MDD share pleiotropic genes (12–20%) and have many common features that make them valuable to examine as a distinct population of interest [14–16,19,20]; and 3) both obesity and MDD and/or anxiety disorders lead to an enormous individual and global burden of disease and disability [1,2]. Therefore, obesity and MDD and/or anxiety disorders may interact thereby augmenting one another’s effect on HR-QoL, and substantially reducing the HR-QoL in people with both exposures.

The interaction between obesity and MDD and/or anxiety disorders on HR-QoL can best be measured by statistical interaction on the additive scale as opposed to conventional interaction [21]. On the one hand, knowledge on interaction effects on the additive scale could provide empirical evidence for public health interventions in vulnerable groups; on the other hand, knowledge on interactions on the multiplicative scale is more relevant in disease etiology [22]. The present study focuses essentially on interaction on the additive scale (i.e. comparing the sum of separate effects of obesity and MDD and/or anxiety versus the combined effect). It is very important to be aware of a slight arbitrariness of interaction on the additive scale from the conventional interaction in terms of defining, detecting and interpreting the interaction effect, particularly for continuous outcomes. In conventional interaction, for instance, it is assumed in advance that obesity modifies the effect of MDD and/or anxiety on poor HR-QoL (i.e. unidirectional). In contrast, the interaction on the additive scale concept considers the potential bidirectional interaction effect of obesity and MDD and/or anxiety on poor HR-QoL, because obesity and MDD and/or anxiety do not precede each other. For instance, if the combined effect of obesity and MDD and/or anxiety disorders surpasses the sum of their separate effects, then intervening on obesity might also reduce the effect of MDD and/or anxiety disorders on poor HR-QoL and vice versa.

Therefore, the aim of this study was to examine the combined effect of obesity and MDD and/or anxiety disorders on HR-QoL and to determine whether the effect of obesity on HR-QoL further increases in persons with and without MDD and/or anxiety disorders. Interaction on the additive scale was used as a measure to test the hypothesis that the combined effect of obesity and MDD and/or anxiety disorders on HR-QoL was larger than the sum of the separate effects.

## Material and Methods

### Study design and population

Data were collected in the ongoing LifeLines Cohort Study, a multi-disciplinary prospective population-based cohort study examining in a unique three-generation design the health and health-related behaviors of 167,729 persons living in the north of The Netherlands. The study employs a broad range of investigative procedures to assess the biomedical, socio-demographic, behavioral, physical and psychological factors which contribute to the health and disease of the general population, with a special focus on multi-morbidity and complex genetics. The design of the LifeLines cohort study has been described elsewhere [23]. For the study, we included N = 89,332 persons, who were enrolled between November 2006 and June 2013. Inclusion criteria for the present study were: age 18 years and older, psychiatric diagnosis, anthropometric measurements, and complete data on HR-QoL.

The LifeLines study protocol was approved by the Ethical Review Board of the University Medical Center Groningen. After receiving full verbal and written information about the study, all participants gave written informed consent. The study was conducted in accordance with the Declaration of Helsinki.

### Measurements

#### General and abdominal obesity

General obesity was assessed using the body mass index (BMI). The BMI was calculated from measured body weight (kg) and height (m). Participants were classified into four BMI classes according to the standard international classification of the World Health Organization (WHO): normal weight (BMI: 18.5–24.99 kg/m^2^), overweight (BMI 25.0–29.99 kg/m^2^), mild obesity (BMI 30.0–34.99 kg/m^2^) and moderate/severe obesity (BMI ≥ 35.0 kg/m^2^). Abdominal obesity was defined using objectively measured waist circumference (WC) of ≥102 cm and ≥88 cm for males and females, respectively [24]. We included abdominal obesity because BMI has been criticized for its inadequate reflection of body composition and we wanted to make our analyses robust. Anthropometric measurements were conducted by nurses during a visit at the LifeLines test location.

#### Major depressive disorders (MDD) and anxiety disorders

MDD and anxiety disorders (generalized anxiety disorder (GAD), social phobia, panic and agoraphobia) were assessed by using the Mini-International Neuropsychiatric Interview (MINI) [25]. The MINI is a short structured diagnostic interview. It is compatible with international diagnostic criteria, including the International Classification of Diseases (ICD-10) and the fourth edition of the Diagnostic and Statistical Manual of Mental Disorders (DSM-IV) [25]. The MINI was designed to meet the need for a short (15 minutes) but accurate structured psychiatric interview for epidemiological studies. The MINI has an excellent inter-rater reliability (Kappa, k > 90), and high retest reliability (k = 0.87 for MDD, k = 0.78 for anxiety disorders) [25]. The MINI interview was conducted by trained interviewers.

#### Health-related quality of Life (HR-QoL)

HR-QoL was assessed by the RAND-36 questionnaire. The RAND-36 is a generic and widely used measure of HR-QoL, designed for use in clinical practice and research, health policy evaluations, and general population surveys [19]. It has been adapted for use in various countries, including the Netherlands [26,27]. The questionnaire covers eight dimensions: Physical Functioning, Role Limitations due to Physical Functioning (Role-Physical), Bodily Pain, General Health, Vitality, Social Functioning, Role Limitations due to Emotional Functioning (Role-Emotional), and Mental Health. Two summary scores of QoL can be calculated: the physical component summary (PCS) and the mental component summary (MCS) scores, reflecting physical and mental QoL, respectively. These summary scores provide information on the patient’s physical and mental QoL in just two values, thereby reducing the number of statistical analyses needed and offering easier interpretation of the data [28]. The PCS- and MCS-scores have good discriminant validity for identifying differences between clinically meaningful groups [29]. The eight domains and two summary scores range from 0 to 100, with higher scores indicating better HR-QoL. PCS- and MCS-scores were computed by using recommended scoring algorithms[30], and standardized by linear z-score transformation to have a mean of 50 and standard deviations of 10 in the US general population.

#### Covariates

Covariates concerned sociodemographic characteristics (age, sex and educational status), lifestyle factors (smoking, alcohol consumption and physical exercise) and common chronic conditions (cardiovascular diseases, hypertension, diabetes, rheumatoid arthritis and cancer). Age was measured in years. Educational level was categorized into low (primary and lower secondary education), middle (higher secondary education) and high education (tertiary or higher education). Physical exercise was defined as the frequency per week in which the respondent typically engaged in moderate physical activities (e.g. walking, bicycling, gardening and household work) for at least half an hour, it was then categorized into high (twice or more per week), medium (once per week) and low (do not exercise/ hardly per week). Smoking status was dichotomized into current smokers and non-smokers. Alcohol intake was assessed based on intake frequency and the average number of units consumed on a drinking day. The number of alcoholic drinks per week was determined by multiplying the number of drinking days per week by the average number of units consumed on a drinking day. The number of alcoholic drinks/week was then divided by 7 to obtain the average number of alcoholic drinks per day. High alcohol consumption was defined as drinking more than an average of 2 drinks of alcohol per day [31]. Common chronic illnesses were assessed by taking the current and/or past history of chronic physical conditions (i.e. cardiovascular diseases, hypertension, diabetes, rheumatoid arthritis and cancer) [26,32].

### Statistical analyses

First, we described participants’ sociodemographics, lifestyle factors, psychopathology and chronic conditions as frequencies, means and standard deviations, using the four BMI categories and abdominal obesity status. The associations of BMI categories and abdominal obesity with MDD and/or anxiety disorders were assessed using binary logistic regression.

Second, we assessed the average deviation in HR-QoL scores for overweight, obese (mild to moderate or severe) persons compared to normal weight persons by MDD and/or anxiety disorders status using one-way analysis of variance (ANOVA). Third, we examined the combined effect of obesity and MDD and/or anxiety disorders on physical and mental QoL. We assessed the presence of interactions by testing the significance of the increment in squared multiple correlation (ΔR^2^) by including the product terms (overweight/obesity x MDD and/or anxiety disorders) in the model adjusted for obesity and MDD and/or anxiety disorders, and also by testing whether the coefficient for the product terms differs from 0 [33]. The regression coefficient of the product term (β) reflects interaction as departure from additivity and is the absolute value difference between the combined effect and the separate effects of obesity and MDD and/or anxiety disorders on physical and mental QoL [21]. The combined effect of obesity and MDD and/or anxiety disorders on physical or mental QoL as measured on a continuous scale is given by the sum of the separate effects of obesity, MDD and/or anxiety disorders and the product term. A synergistic effect of obesity and MD/anxiety on physical and mental QoL is reflected as B < 0, while B > 0 represents a negative interaction (antagonistic) of obesity and MDD and/or anxiety disorders on physical and mental QoL:B = 0 represents no interaction effect of obesity and MDD and/or anxiety disorders on physical and mental QoL.

Fourth, using linear regression models, we examined the association of BMI categories with physical and mental QoL in persons with and without MDD and/or anxiety disorders. In these analyses, we tested four different models of general and abdominal obesity to adjust for other variables potentially affecting the associations of obesity and/or MDD and/or anxiety disorders with physical and mental QoL. Model 1 tested the crude association of overweight and obesity categories with physical and mental QoL compared to normal weight category, and stratified by MDD and/or anxiety disorders status. Model 2 adjusted additionally for socio-demographic factors (i.e. age, sex and education). In Model 3, lifestyle factors (i.e. physical exercise, smoking and alcohol) were added, and Model 4 contained all variables from Model 3 plus major chronic conditions (i.e. cardiovascular diseases, hypertension, diabetes, rheumatoid arthritis and cancer).

All analyses were performed using SPSS statistical software (SPSS version 22.0), a two-sided p<0.05 was considered statistically significant.

## Results

### Sample characteristics

Table 1 shows the characteristics of participants (N = 89,332) by BMI categories and abdominal obesity status. The prevalences of overweight, mild obesity, moderate/severe obesity and abdominal obesity were 40%, 12%, 4% and 36%, respectively, and of MDD and/or anxiety disorders was 11%. In addition, mild, moderate/severe and abdominal obesity were associated with MDD and/or anxiety disorders (odds ratio, OR (95% confidence interval, CI): = 1.20 (1.12; 1.28), 1.81 (1.65; 1.99) and 1.40 (1.34; 1.46)), respectively. Overweight was not associated with MDD/anxiety disorders OR = 0.96 (0.91; 1.01).

**Table 1:** LifeLines cohort characteristics by BMI categories and abdominal obesity.

		BMI categories				Abdominal
Characteristics	Total N = 89,332	Normal weight n = 39960 (44.7%)	Over-weight n = 35355 (39.6%)	Mild obesity n = 10551 (11.8%)	Moderate/ severe obesity n = 3466 (3.9%)	obesity^a^ n = 32231 (36.1%)
Age, mean (SD)	44.4(12.3)	41.8 (12.3)	46.5 (12.0)	47.0 (11.7)	45.3 (11.0)	47.6 (11.8)
Sex, females, (%)	58.6	65.5	49.3	57.9	75.2	70.7
Educational status, (%)						
High	29.7	35.7	27.1	20.2	16.0	22.1
Middle	40.2	40.6	39.6	39.7	41.8	39.3
Low	30.1	23.7	33.4	40.1	42.2	38.6
Frequency of exercise, (%)						
High (≥ 2x/wk)	86.8	87.9	87.0	84.0	79.5	84.6
Medium (1x/wk)	8.6	8.0	8.7	10.0	11.5	9.7
Low (No or hardly/wk)	4.6	4.1	4.3	6.0	9.0	5.7
Current smokers, (%)	21.5	22.5	21.1	19.8	18.6	20.3
High alcohol consumption, (%)	8.0	6.8	9.5	8.7	5.3	7.5
Major depression, (%)	2.2	2.0	1.9	3.1	5.7	3.0
Anxiety disorders, (%)	9.9	9.6	9.3	11.1	15.5	11.8
Common chronic conditions						
Hypertension, (%)	25.3	15.9	28.1	41.8	51.7	37.1
Cardiovascular diseases, (%)	3.3	2.4	3.6	4.6	6.1	4.6
Diabetes, (%)	2.3	0.8	2.3	5.4	9.1	4.4
Rheumatoid arthritis, (%)	3.2	2.4	3.2	5.0	8.5	5.2
Cancer, (%)	4.4	4.0	4.7	4.6	4.5	5.4
HR-QoL (PCS, MCS)						
PCS, mean (SD)	51.3 (7.2)	52.3 (6.6)	51.2 (7.1)	49.2 (8.3)	46.8 (9.4)	49.6 (8.1)
MCS, mean (SD)	52.6 (8.4)	52.2 (8.4)	53.1 (8.1)	52.7 (8.8)	51.8 (9.8)	52.3 (7.2)

HR-QoL: Health-related quality of life; PCS: physical component summary score; MCS: mental component summary score; Normal weight (BMI: 18.5–24.99 kg/m^2^), overweight (BMI 25.0–29.99 kg/m^2^), mild obesity (BMI 30.0–34.99 kg/m^2^) and moderate/severe obesity (BMI ≥ 35.0 kg/m^2^)

^a^ Waist circumference (WC) ≥ 102 cm for males and ≥88cm for females.

In Table 2 we present the average deviation in HR-QoL domains in participants with obesity and MDD and/or anxiety disorders compared with normal weight counterparts. On all physical health measures and health perceptions overweight and obese persons had a significantly poorer HR-QoL than normal weight persons (Table 2). However, overweight and normal weight participants showed no difference in psychosocial aspects of HR-QoL (social functioning and emotional role) (p<0.01).

**Table 2:** Health-related quality of life (HR-QoL) domains in participants by BMI categories and MDD and/or anxiety disorders: deviation with standard error from the mean for normal weight participants.

	No MDD/anxiety disorder (n = 79820)				Anxiety alone (n = 8838)				Major depression alone (n = 2004)				MDD/anxiety disorder (n = 9512)			
	Normal weight, mean (SD)^a^	Over-weight	Mild obesity	Moderate/ Severe obesity	Normal weight, mean (SD)^a^	Over-weight	Mild obesity	Moderate/ Severe obesity	Normal weight	Over-weight	Mild obesity	Moderate obesity	Normal weight, mean (SD)^a^	Over-weight	Mild obesity	Moderate/ Severe obesity
Physical function	93.6 (11.2)	**-2.7 (0.1)**	**-7.2 (0.2)**	**-13.7 (0.3)**	82.5 (24.8)	**-3.8 (0.4)**	**-10.8 (0.6)**	**-18.3 (0.8)**	**82.0 (19.3)**	**-5.2 (1.1)**	**-14.6 (1.4)**	**-21.4 (1.7)**	87.6 (15.9)	**-3.8 (0.4)**	**-11.1 (0.6)**	**-19.4 (0.8)**
Role-physical	89.7 (25.9)	**-1.2 (0.2)**	**-5.3 (0.3)**	**-9.0 (0.5)**	77.2 (34.5)	**-4.0 (0.9)**	**-11.4 (1.3)**	**-15.9 (1.8)**	**58.0 (43.5)**	**-6.0 (2.3)**	**-12.1 (2.9)**	**-18.6 (3.5)**	73.9 (38.0)	**-3.9 (0.9)**	**-11.5 (1.3)**	**-17.4(1.7)**
Role-emotional	93.9 (20.2)	0.2 (0.2)	**-1.3 (0.2)**	**-2.5 (0.4)**	72.9 (38.9)	**2.0 (1.0)**	-2.6 (1.4)	**-9.1 (1.9)**	**38.7 (42.9)**	**0.2 (2.2)**	**-0.5 (2.8)**	**-2.6 (3.4)**	64.9 (42.2)	**2.0 (1.0)**	**-3.0 (1.4)**	**-9.4 (1.9)**
Social functioning	89.9 (15.5)	0.2 (0.1)	**-1.4 (0.2)**	**-3.6 (0.3)**	64.5 (22.4)	0.5 (0.6)	**-2.7 (0.8)**	**-8.3 (1.2)**	**51.1 (25.3)**	**0.7 (1.3)**	**0.3 (1.6)**	**-6.8 (2.0)**	69.7 (24.7)	0.6 (0.6)	**-2.7 (0.8)**	**-8.7 (1.1)**
Bodily pain	86.9 (17.2)	**-1.6 (0.1)**	**-4.9 (0.2)**	**-8.7 (0.4)**	69.5 (26.0)	**-2.0 (0.5)**	**-7.0 (0.8)**	**-11.6 (1.0)**	**68.2 (24.8)**	**-4.0 (1.3)**	**-8.3 (1.7)**	**-14.2 (2.0)**	76.5 (21.7)	**-2.0 (0.5)**	**-6.9 (0.7)**	**-12.1 (1.0)**
Mental health	81.1 (11.7)	**1.1 (0.1)**	**0.7 (0.1)**	**-0.5 (0.2)**	58.2 (15.3)	**1.3 (0.4)**	0.3 (0.6)	**-3.6 (0.8)**	**48.2 (18.1)**	**0.7 (0.9)**	**1.0 (1.1)**	**-2.1 (1.4)**	63.2(17.9)	**1.3 (0.4)**	0.1 (0.6)	**-3.8 (0.8)**
Vitality	69.6 (15.4)	**0.2 (0.1)**	**-2.2 (0.2)**	**-5.4 (0.3)**	54.6 (15.5)	0.1 (0.4)	**-2.4 (0.6)**	**-7.1 (0.9)**	**38.4 (17.1)**	**-0.3 (0.8)**	**-1.9 (1.1)**	**-5.1 (1.3)**	52.7 (18.9)	-0.2 (0.4)	**-2.8 (0.6)**	**-7.4 (0.8)**
General health	69.7 (11.6)	**-0.6 (0.1)**	**-1.8 (0.1)**	**-4.5 (0.2)**	58.6 (12.1)	**-1.3 (0.3)**	**-3.8 (0.5)**	**-7.2 (0.7)**	**58.2 (15.7)**	**-2.2 (0.8)**	**-3.1 (1.0)**	**-8.1 (1.2)**	63.2 (14.3)	**-1.2 (0.3)**	**-3.7 (0.5)**	**-7.5 (0.6)**
PCS	52.4 (6.3)	**-1.0 (0.1)**	**-2.8 (0.1)**	**-5.0 (0.1)**	49.6 (10.1)	**-1.8 (0.2)**	**-4.5 (0.3)**	**-6.6. (0.4)**	**49.9 (10.1)**	**-2.5 (0.6)**	**-5.6 (0.7)**	**-8.5 (0.8)**	51.1 (8.6)	**-1.8 (0.2)**	**-4.5 (0.3)**	**-7.0 (0.4)**
MCS	53.4 (6.8)	**0.7 (0.1)**	**0.7 (0.1)**	**0.6 (0.1)**	41.3 (11.1)	**1.3 (0.3)**	**0.9 (0.4)**	-1.0 (0.6)	32.3 (12.0)	1.2 (0.6)	2.3 (0.8)	1.2 (0.9)	42.1 (12.4)	**1.3 (0.3)**	**0.8 (0.4)**	-1.0 (0.5)

^**a**^ Reference group

SD: standard deviation; MDD: major depressive disorder; HR-QoL: Health related quality of life; PCS: physical component summary score; MCS: Mental component summary score; Normal weight (BMI: 18.5–24.99kg/m2), overweight (BMI 25.0–29.99 kg/m2), mild obesity (BMI 30.0–34.99 kg/m2) and moderate/severe obesity (BMI ≥ 35.0 kg/m2); **Bold figures** reflect statistically significant deviations from the mean for normal weight participants (p<0.05).

### The combined effect of obesity and MDD and/or anxiety disorders on HR-QoL

Table 3 shows the interaction of obesity and major depression/anxiety with physical and mental quality of life, which is the main finding of this study. We found that the combined effect of obesity and MDD and anxiety on physical QoL was (B = -1.32, 95%CI: -1.75; -0.90 and B = -1.27, 95%CI: -1.73; -0.81, respectively (Table 3). The interaction effect sizes of B = -1.32 indicated that the combined effect of obesity and MDD and/or anxiety disorders on physical QoL was greater than the additive effect. However, the combined effect of obesity and major depression alone on mental QoL was less than the additive effect (Table 3).

**Table 3:** The separate association of obesity, major depression/anxiety and their interactions with physical and mental quality of life.

Parameter	Physical QoL (PCS-score)		Mental QoL (MCS-score)	
	Crude estimate	Adjusted estimate	Crude estimate	Adjusted
	B(95%-CI)	B(95%-CI)^a^	B(95%-CI)	B(95%-CI)^a^
Obesity	**-2.96 (-3.09; -2.83)**	**-2.25 (-2.41; -2.10)**	**0.20 (0.05; 0.34)**	**0.29 (0.12; 0.45)**
Major depression alone	**-1.50 (-1.68; -1.32)**	**-1.10 (-1.33; -0.88)**	**-10.10 (-10.31; -9.90)**	**-9.85 (-10.09; -9.60)**
Obesity x Major depression	**-1.28 (-1.65; -0.92)**	**-1.32 (-1.75; -0.90)**	**0.57 (0.18, 0.97)**	**0.59 (0.13; 1.05)**
Obesity	**-2.89 (-3.03; -2.76)**	**-2.20 (-2.36; -2.04)**	**0.23 (0.08; 0.38)**	**0.29 (0.12; 0.46)**
Anxiety alone	**-1.50 (-1.67; -1.32)**	**-1.23 (-1.43; -1.03)**	**-10.61 (-10.80; -10.42)**	**-9.81 (-10.03; -9.59)**
Obesity x anxiety	**-1.43 (-1.83; -1.03)**	**-1.27 (-1.73; -0.81)**	**-0.50 (-0.93; -0.07)**	**-0.60 (-1.09; -0.10)**
Obesity	**-2.84 (-2.98; -2.70)**	**-2.17 (-2.33; 2.01)**	**0.34 (0.20; 0.50)**	**0.37 (0.20; 0.54)**
Major depression/anxiety	**-0.80 (-0.89; -0.72)**	**-0.64 (-0.74; -0.54)**	**-5.52 (-5.61; -5.43)**	**-5.12 (-5.23; -5.02)**
Obesity x Major depression/anxiety	**-0.82 (-1.01; -0.62)**	**-0.70 (-0.92; -0.48)**	**-0.35 (-0.56; -0.15)**	**-0.40 (-0.63; -0.16)**

*Abbreviations*: PCS: physical component summary score; MCS: Mental component summary score; Obesity (BMI ≥ 30.0 kg/m2) compared to non-obese.

^a^ Adjusted for age, sex, educational status, smoking, exercise, alcohol consumption and major chronic conditions (i.e. cardiovascular diseases, hypertension, diabetes, rheumatoid arthritis and cancer). For instance, the combined effect of obesity and major depression on PCS-score was = -2.96–1.50–1.28 = -5.74, while the sum of individual effects of obesity and major depression/anxiety assuming no interaction was = -2.96–1.50 = -4.46. The departure from additivity is given by = -5.74- (-4.46) = -1.28, by definition, the regression coefficient of the product term.

Furthermore, our stratified analysis revealed a significant association of physical QoL across BMI categories and abdominal obesity in depressed and anxious individuals (Table 4). However, the association of obesity and mental QoL was not statistically significant in depressed and anxious individuals in the general population. In non-depressed and anxious individuals, obesity was significantly associated with better mental QoL (Table 5).

**Table 4:** Crude and adjusted regression coefficients (B) for physical quality of life with general and abdominal obesity categories, stratified by major depression/anxiety.

Mental disorder and body weight	N (%)	Physical component summary score of HR-QoL			
		Model 1	Model 2	Model 3	Model 4
		B (95%-CI)	B (95%-CI)	B (95%-CI)	B (95%-CI)
No major depression/anxiety (n = 79,820)					
Normal weight	35854 (44.9)	Reference	Reference	Reference	Reference
Overweight	31833 (39.9)	**-1.07 (-1.19; -0.95)**	**-0.74 (-0.86; -0.61)**	**-0.73 (-0.85; -0.60)**	**-0.64 (-0.76; -0.52)**
Mild obesity	9266 (11.6)	**-2.87 (-3.05; -2.69)**	**-2.37 (-2.55; -2.19)**	**-2.34 (-2.52; -2.16)**	**-1.87 (-2.05; -1.70)**
Moderate/severe obesity	2867 (3.6)	**-5.05 (-5.35; -4.75)**	**-4.49 (-4.79; -4.19)**	**-4.44 (-4.73; -4.14)**	**-3.55 (-3.84; -3.26)**
No abdominal obesity	51708 (64.8)	Reference	Reference	Reference	Reference
Abdominal obesity	28112 (35.0)	**-2.36 (-2.48; -2.25)**	**-1.75 (-1.87; -1.64)**	**-1.72 (-1.84; -1.60)**	**-1.37 (-1.49; -1.26)**
Anxiety alone (n = 8838)					
Normal weight	3832 (43.4)	Reference	Reference	Reference	Reference
Overweight	3298 (37.3)	**-1.90 (-1.94; -0.94)**	**-1.44 (-1.94; -0.94)**	**-1.44 (-1.94; -0.94)**	**-1.29 (-1.79, -0.78)**
Mild obesity	1172 (13.3)	**-4.37 (-4.34; -2.95)**	**-3.65 (-4.34; -2.95)**	**-3.65 (-4.35; -2.95)**	**-3.20 (-3.90; -2.49)**
Moderate/severe obesity	536 (6.1)	**-6.68 (-6.97; -5.07)**	**-6.03 (-6.97; -5.07)**	**-5.94 (-6.89; -4.99)**	**-5.30 (-6.27; -4.33)**
No abdominal obesity	5047 (57.1)	Reference	Reference	Reference	Reference
Abdominal obesity	3791 (42.9)	**-3.32 (-3.77; -2.87)**	**-2.59 (-3.06; -2.13)**	**-2.57 (-3.03; -2.11)**	**-2.19 (-2.66; -1.72)**
Major depression alone (n = 2004)					
Normal weight	808 (40.3)	Reference	Reference	Reference	Reference
Overweight	673 (33.6)	**-2.21 (-3.54; -0.88)**	**-1.61 (-2.95; -0.27)**	**-1.64 (-2.98; -0.30)**	**-1.50 (2.84; -0.15)**
Mild obesity	326 (16.3)	**-5.69 (-7.34; -4.05)**	**-4.92 (-6.57; -3.27)**	**-4.89 (-6.54; -3.23)**	**-4.60 (-6.29; -2.91)**
Moderate/severe obesity	197 (9.8)	**-8.07 (-10.01; -6.13)**	**-7.47 (-9.40; -5.53)**	**-7.36 (-9.29; -5.42)**	**-6.88 (-8.88; -4.89)**
No abdominal obesity	1050 (52.4)	Reference	Reference	Reference	Reference
Abdominal obesity	954 (47.6)	**-4.31 (-5.45; -3.16)**	**-3.75 (-4.92; -2.58)**	**-3.74 (-4.91; -2.56)**	**-3.33 (-4.54; -2.12)**
Major depression/anxiety (n = 9,512)					
Normal weight	4106 (43.2)	Reference	Reference	Reference	Reference
Overweight	3522 (37.0)	**-1.87 (-2.36; -1.38)**	**-1.40 (-1.89; -0.90)**	**-1.40 (-1.89; -0.91)**	**-1.24 (-1.74; -0.75)**
Mild obesity	1285 (13.5)	**-4.35 (-5.02; -3.67)**	**-3.64 (-4.32; -2.97)**	**-3.62 (-4.30; -2.95)**	**-3.15 (-3.84; -2.47)**
Moderate/severe obesity	599 (6.3)	**-6.97(-7.89; -6.05)**	**-6.30 (-7.21; -5.39)**	**-6.20 (-7.11; -5.28)**	**-5.53 (-6.46; -4.60)**
No abdominal obesity	5393 (56.7)	Reference	Reference	Reference	Reference
Abdominal obesity	4119 (43.3)	**-3.39 (-3.84; -2.23)**	**-2.68 (-3.13; -2.23)**	**-2.64 (-3.09; -2.19)**	**-1.99 (-2.43; -1.56)**

*Abbreviations*: HR-QoL: Health related quality of life; Normal weight (BMI: 18.5–24.99 kg/m2), overweight (BMI 25.0–29.99 kg/m2), mild obesity (BMI 30.0–34.99 kg/m2) and moderate/severe obesity (BMI ≥ 35.0 kg/m2); B; regression coefficient; 95%-CI: 95%-confidence interval; Model 1: Crude estimate; Model 2: Model 1 plus sociodemographic factors (i.e. Age, sex, educational status); Model 3: Model 2 plus lifestyle factors (i.e. smoking, exercise, alcohol consumption); Model 4: Model 3 plus major chronic conditions (i.e. cardiovascular diseases, hypertension, diabetes, rheumatoid arthritis and cancer); **Bold figures** reflect statistically significant estimates (p<0.05).

**Table 5:** Crude and adjusted regression coefficients (B) for mental quality of life with general and abdominal obesity categories stratified by major depression/anxiety.

Mental disorder and body weight		Mental component summary score (MCS) of HR-QoL			
				
	N (%)	Model 1	Model 2	Model 3	Model 4
		B (95%-CI)	B (95%-CI)	B (95%-CI)	B (95%-CI)
No major depression/anxiety (n = 79,820)					
Normal weight	35854 (44.9)	Reference	Reference	Reference	Reference
Overweight	31833 (39.9)	**0.67 (0.55; 0.79)**	0.11 (-0.01; 0.23)	**0.12 (-0.01; 0.24)**	**0.19 (0.07; 0.31)**
Mild obesity	9266 (11.6)	**0.66 (0.48; 0.84)**	**0.19 (0.01; 0.37)**	**0.20 (0.01; 0.38)**	**0.38 (0.20; 0.56)**
Moderate/severe obesity	2867 (3.6)	**0.58 (0.29; 0.88)**	**0.49 (0.20; 0.80)**	**0.48 (0.18; 0.77)**	**0.77 (0.47; 1.06)**
No abdominal obesity	51708 (64.8)	Reference	Reference	Reference	Reference
Abdominal obesity	28112 (35.0)	**0.22 (0.11; 0.34)**	**0.19 (0.07; 0.31)**	**0.20 (0.08; 0.32)**	**0.34 (0.22; 0.45)**
Anxiety alone (n = 8838)					
Normal weight	3832 (43.4)	Reference	Reference	Reference	Reference
Overweight	3298 (37.3)	4.05 (-6.10; 12.17)	3.04 (-6.10; 12.17)	5.59 (-3.08; 14.27)	2.75 (-6.74; 12.25)
Mild obesity	1172 (13.3)	2.84 (-7.91; 13.58)	0.91 (-10.38; 12.20)	8.10 (-3.14; 19.35)	4.90 (-7.18; 16.99)
Moderate/severe obesity	536 (6.1)	**-18.73 (-34.58; -2.88)**	**-20.02 (-36.19; -3.85)**	-10.90 (-26.95; 5.15)	-16.89 (-36.07; 2.30)
No abdominal obesity	5047 (57.1)	Reference	Reference	Reference	Reference
Abdominal obesity	3791 (42.9)	0.71 (0.10; 1.31)	-0.10 (-0.71; 0.53)	-0.14 (-0.76; 0.48)	0.18 (-0.45; 0.82)
Major depression alone (n = 2004)					
Normal weight	808 (40.3)	Reference	Reference	Reference	Reference
Overweight	673 (33.6)	-3.08 (-20.87; 14.72)	-5.68 (-26.72; 15.37)	-	**-**
Mild obesity	326 (16.3)	17.67 (-2.51; 37.85)	12.89 (-8.15; 33.94)	-	**-**
Moderate/severe obesity	197 (9.8)	-14.09 (-34.27; 6.09)	-10.45 (-29.52; 8.62)	-	**-**
No abdominal obesity	1050 (52.4)	Reference	Reference	Reference	Reference
Abdominal obesity	954 (47.6)	**1.51 (0.27; 2.76)**	0.88 (-0.41; 2.16)	0.86 (-0.43; 2.15)	0.95 (-0.38; 2.28)
Major depression/anxiety (n = 9,512)					
Normal weight	4106 (43.2)	Reference	Reference	Reference	Reference
Overweight	3522 (37.0)	**1.34 (0.69; 2.00)**	**0.89 (0.23; 1.55)**	**0.83 (0.17; 1.49)**	**0.98 (0.32; 1.64)**
Mild obesity	1285 (13.5)	**0.95 (0.05; 1.85)**	0.45 (-0.3446; 1.35)	0.31 (-0.60; 1.21)	0.71 (-0.21; 1.63)
Moderate/severe obesity	599 (6.3)	**-1.56 (-2.79; -0.59)**	**-1.82 (-3.04;-0.59)**	**-1.90 (-3.12; -0.68)**	**-1.33 (-2.58; -0.09)**
No abdominal obesity	5393 (56.7)	Reference	Reference	Reference	Reference
Abdominal obesity	4119 (43.3)	**0.63 (0.05; 1.22)**	-0.06 (-0.66; 0.54)	-0.11 (-0.71; 0.49)	0.23 (-0.39; 0.84)

*Abbreviations*: HR-QoL: Health related quality of life; Normal weight (BMI: 18.5–24.99kg/m2), overweight (BMI 25.0–29.99 kg/m2), mild obesity (BMI 30.0–34.99 kg/m2) and moderate/severe obesity (BMI ≥ 35.0 kg/m2); B; regression coefficient;95%-CI: 95%-confidence interval; Model 1: Crude estimate; Model 2: Model 1 plus sociodemographic factors (i.e. Age, sex, educational status); Model 3: Model 2 plus lifestyle factors (i.e. smoking, exercise, alcohol consumption); Model 4: Model 3 plus major chronic conditions (i.e. cardiovascular diseases, hypertension, diabetes, rheumatoid arthritis and cancer); **Bold figures** reflect Statistically significant estimates (p<0.05).

## Discussion

In this large, representative cohort study, we found that the combined effects of obesity and MDD and/or anxiety disorders on physical and mental QoL were greater than the sum of their separate effects. Moreover, general and abdominal obesity were significantly associated with a poorer physical QoL in persons with and without MDD and/or anxiety disorders after adjustment for potential confounders. General and abdominal obesity were found to be associated with better mental QoL in persons without MDD/anxiety disorders. This association was not found in persons with MDD/anxiety disorders after adjustment for potential confounders.

The combined effect of obesity and MDD and/or anxiety disorders on physical QoL was significantly larger than the sum of their estimated separate effects. As indicated in the interaction model, the average physical QoL for obese persons (BMI≥30) with MDD was 4.46 points lower than that of non-obese non-depressed persons. With sufficient-cause interaction in mind, obesity and MDD and/or anxiety disorders are component causes that act in concert and are associated with poor physical QoL. Obesity may interact with MDD and/or anxiety disorders, whereby each augmenting the effect of the other on physical QoL. Although this finding is based on cross-sectional data, it seems plausible because obesity shares genetic and complex biologic etiologic substrates with MDD and/or anxiety disorders [14,20,34]. Although the presence of inflammatory responses and the crucial role of cytokines have been established more for depression than for anxiety [35], the joint presence of MDD/anxiety disorders and obesity can contribute to morbidity and poorer physical conditions [14,20,36]. In addition, subtypes of both obesity and MDD and/or anxiety disorders are assumed to be related to stress, which is characterized by endogenous overproduction of adrenocorticotrophin (ACTH) and cortisol [37] This chronic hypercortisolism induces (abdominal) obesity and depressive symptoms, and severely reduced HR-QoL, conditions which are all known to improve in the majority of patients after treating the disease by surgery or medication [37]. Hence, the current study shows that joint exposure of obesity and MDD and/or anxiety disorders decreases physical QoL, and the joint association of obesity and MDD and/or anxiety disorders is greater than the additive effect.

The combined effect of obesity and MDD/anxiety disorders on mental QoL was also significantly greater than the sum of their separate effects. The interaction model indicates that mean mental QoL scores were 5.52 points lower in obese persons (BMI≥30) with MDD/anxiety disorders than in those without such disorders. This underlines the findings of Atlantis et al [38], who reported mean mental QoL scores of 39% to 43% points lower in all BMI groups with MDD compared to groups with normal weight and without MDD. Although the excess reduction of the mental QoL score is expected to be related to MDD/anxiety disorders, being obese may further decrease mental QoL. In Western societies, where being thin is considered attractive beauty, obesity might impact on an individual’s body image and self-esteem, and thus be a source of clinically significant distress or depression, thereby reducing the quality of life [39]. Hilbert et al have also shown that obese persons taking part in social activities face more stigma and prejudice than do obese persons [40].

Furthermore, multivariate analyses showed that general and abdominal obesity were consistently associated with a poorer physical QoL in persons both with and without MDD and/or anxiety disorders. This finding is in line with a systematic review and several other studies [17, 41–50], which found a robust relationship between overweight, obesity and poorer HR-QoL. The severity of obesity and treatment-seeking behavior might be underlying factors linking obesity and poor physical QoL [17]. Higher body weight and an excess of visceral fat are associated with higher rates of health care utilization [17]. Persons who had sought treatment or tried to lose weight were significantly more impaired on physical measures (e.g. bodily pain, general health and vitality) of HR-QoL than those who did not try to lose weight [17]. As presented in this study, the physical measures of HR-QoL showed significant linear reductions in obese persons, and the greatest decline (20 point lower) was observed in obese persons with MDD and/or anxiety disorders (Table 2). The effect of obesity on mental QoL seems reduced with increasing obesity in anxious individuals compared to depressed counterparts, but was not statically significant after adjustment of potential confounders (Table 2 and Table 5). Therefore, all forms of obesity are associated with poorer physical QoL, and the associations may even be stronger in obese persons who also have MDD and/or anxiety disorders.

We did not find associations of general and abdominal obesity with mental QoL in persons with MDD and/or anxiety disorders. The associations were explained by lifestyle factors and chronic conditions. However, in persons without MDD and/or anxiety disorders, both general and abdominal obesity were significantly associated with a better mental QoL after adjustment for potential confounders. This finding contradicts those of Cameron et al [41] and Vetter et al [44], who found that BMI change was associated with a decrease in mental QoL. Laaksonen et al and Renzaho et al reported no association between obesity and mental QoL and other mental health domains [43,45]. Possible explanations for these discrepancies are differences in the assessment of MDD and anxiety disorders, the study population and confounding factors. Most of the previous studies used self-reported measures of MDD and/or anxiety disorders [37,43], while the present study used a psychiatric interview. In the previous studies, the potential interactions between obesity and MDD and/or anxiety disorders were also not considered. Generally, mental health problems could be expected in obese persons because of the stigma associated with excess body weight, but some obese persons appear to have essentially normal psychosocial functioning. This might be due to several underlying factors, such as perceived body image, self-esteem, and the level of severity and persistence of depressive disorders in obese persons [39, 43]. It could also be due to the generic instrument used to assess mental Qol, where the generic versions may not reflect the impact of weight related stigma and discrimination issues on mental QoL in obese persons. Taken together, these results indicate that the joint exposures of obesity and MDD and/or anxiety disorders are associated with poorer physical and mental QoL; obesity alone has no effect on mental QoL in the general population.

### Strengths and limitations

The major strength of our study is the nature of the study population, which is derived from the general population and both large and well characterized. The sample size of N = 89,332 participants allowed us to perform subgroup analysis with different BMI categories and MDD and/or anxiety disorders status. Moreover, a comprehensive assessment of chronic conditions, a psychiatric interview, and two anthropometry metrics, i.e. BMI and WC, were used. The similar results for BMI and WC suggest that our results are robust.

The main limitation of our study is its cross-sectional nature, i.e. inferences concerning the direction of the observed associations between obesity, MDD and/or anxiety disorders and HR-QoL cannot be made. Moreover, the use of the RAND-36 may be a limitation, as many researchers for its inadequate reflection of HR-QoL have criticized it. The RAND-36 may also not cover all essential health aspects pertinent to a particular disease. However, it does have the advantage of enabling HR-QoL comparisons across different diseases. It has been shown to have had a high degree of responsiveness to diseases, by which it discriminates between people in different categories of overweight and obesity, as presented here. Nevertheless, it is highly important to sue a multidimensional instrument embracing different health aspects that do not necessarily correlate to each other, such as obesity specific measures like the impact of weight on quality of life (IWQoL-Lite). Furthermore, misclassification may have occurred in the measurements of smoking and alcohol consumption, which were based on self-administered questionnaires. However, earlier studies have showed that self-reported smoking status and alcohol consumption can be used with notable confidence and provide an estimate comparable to the actual consumption [32, 49]. Finally, because data on the binge eating disorder (BED) was not available we were not able to analyse the potential role of this variable in the association of obesity and MDD and/or anxiety with poor HR-QoL. Further studies are needed to assess the potential role of BED.

### Practical and policy implications

In this large, representative study, we showed that in the general population the combination of obesity and MDD and/or anxiety disorders is associated with a poor HR-QoL. This combined effect may have implications for prevention and public health measures if confirmed in prospective studies. The magnitude of the impact of obesity and MDD and/or anxiety disorders on a range of HR-QoL dimensions indicates that the successful management of depression in the primary care setting would result in a significant alleviation of suffering in obese adults. In light of this, the weight-increasing side effects of many commonly used antidepressants should also be considered. Where possible, a more restrained use of those antidepressants with the greatest weight stimulating effects would seem advisable, in particular for obese persons [50]. Several studies have shown that a weight loss program can lead to a significant reduction in depression scores [51]. Thus, monitoring depressive and anxiety symptoms is important in obese persons, and maintaining normal weight or reducing excess weight would be by far the best approach to improve the HR-QoL.

## Conclusions

In conclusion, the combined effect of obesity and MDD and/or anxiety disorders on HR-QoL is greater than the sum of the separate effects of obesity and MDD and/or anxiety disorders on HR-QoL. Moreover, both general and abdominal obesity are associated with poor physical QoL. General and abdominal obesity without MDD and/or anxiety disorders are associated with better mental QoL. Longitudinal studies are needed to explore the causal pathways between obesity, MDD and/or anxiety disorders and HR-QoL.
